# Supplementary material for: RevGel-seq: instrument-free single-cell RNA sequencing using a reversible hydrogel for cell-specific barcoding
Source: Sci Rep. 2023 Mar 24;13:4866. doi: 10.1038/s41598-023-31915-y (PMC10039079; doi:10.1038/s41598-023-31915-y)
Supplement: Supplementary file 1 — Supplementary Information. [file 41598_2023_31915_MOESM1_ESM.pdf]

Supplementary Information

RevGel-seq: instrument-free single-cell RNA sequencing using a reversible hydrogel for cell-specific barcoding.

Jun Komatsu, Alba Cico, Raya Poncin, Maël Le Bohec, Jörg Morf, Stanislav Lipin, Antoine Graindorge, Hélène Eckert, Azadeh Saffarian, Léa Cathaly, Frédéric Guérin, Sara Majello, Damien Ulveling, Anaïs Vayaboury, Nicolas Fernandez, Dilyana Dimitrova, Xavier Bussell, Yannick Fourne, Pierre Chaumat, Barbara André, Elodie Baldivia, Ulysse Godet, Mathieu Guinin, Vivien Moretto, Joy Ismail, Olivier Caille, Natacha Roblot, Carine Beaupère, Alexandrine Liboz, Ghislaine Guillemain, Bertrand Blondeau, Pierre Walrafen,*, Stuart Edelstein*

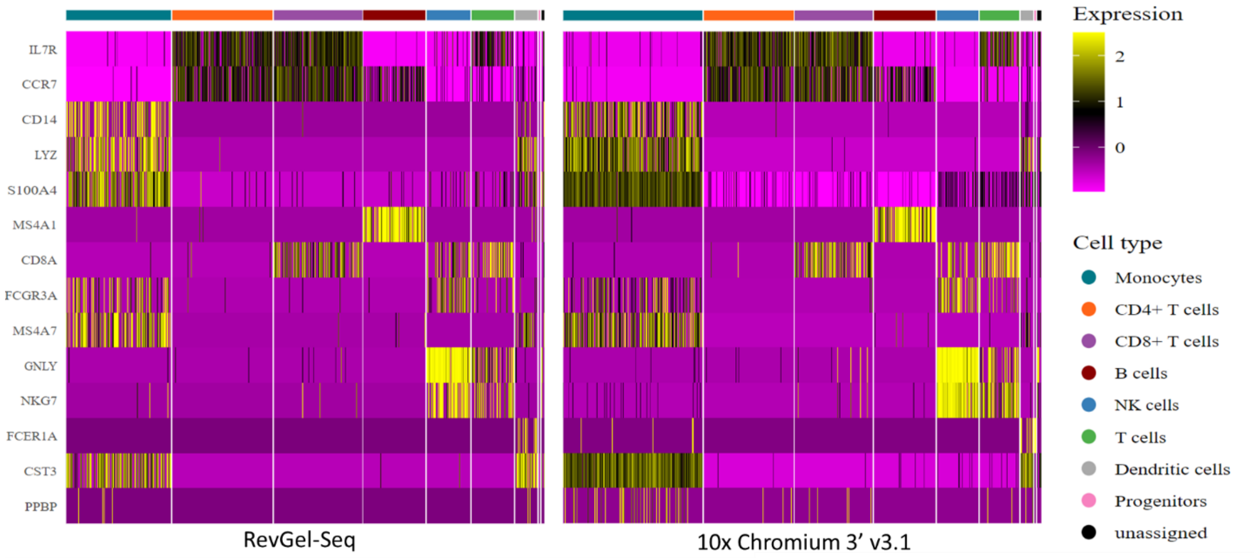

Figure S1. Heatmap of the human PBMC sample prepared with RevGel-seq (left) and with 10x Chromium 3' v3.1 (right), showing the log-normalized expression of 14 typical canonical PBMC marker genes (shown on the left) in each analyzed cell, where cells are grouped by automatically annotated cell type (8 subtypes, shown on the right) based on the reference dataset MonacoImmuneData. Marker genes: IL7R, CCRT for T cells; CD14, LYZ, S100A4, FCGR3A, MS4A7 for Monocytes; CD8A for CD8+ T cells; MS4A1 for B cells; GNLY, NKG7 for Natural Killer (NK) cells; FCER1A and CST3 for Dendritic cells; PPBP for Platelet cells).

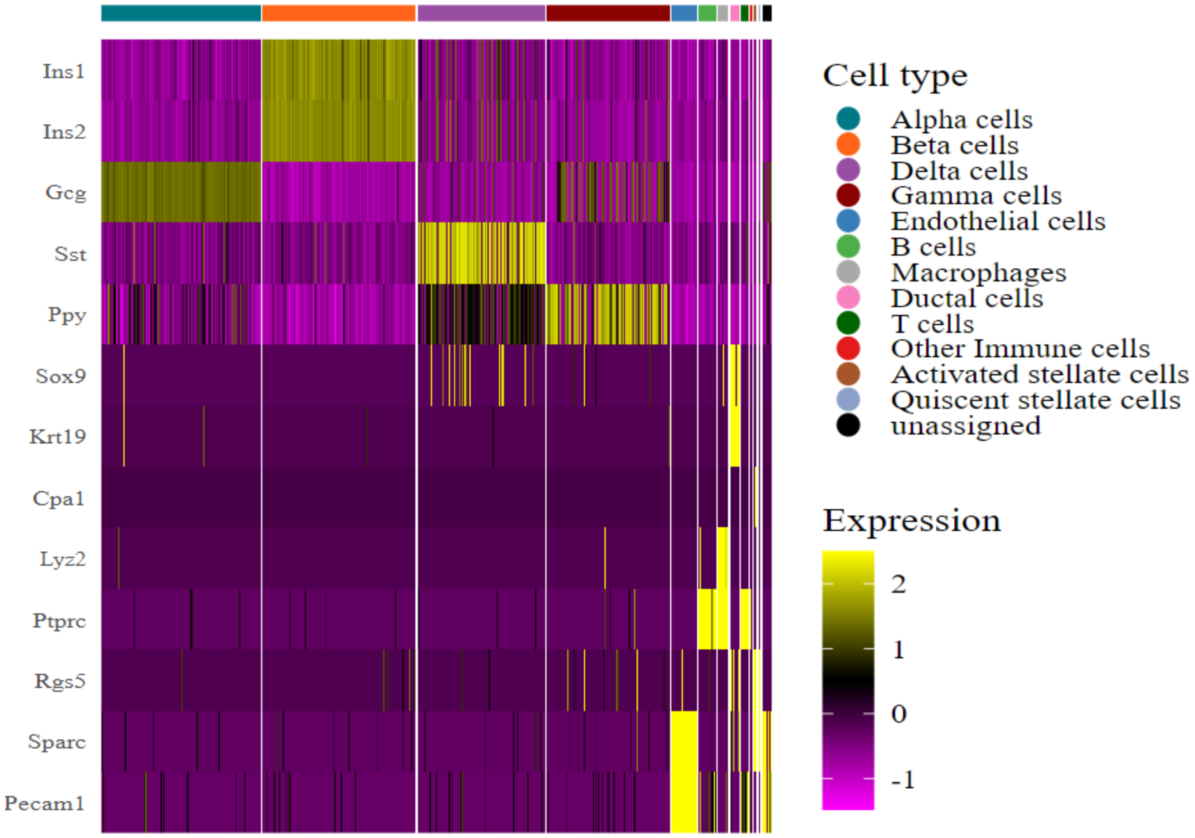

Figure S2. Heatmap of the pancreatic islet cells sample prepared with RevGel-seq, showing the log-normalized expression of 13 marker genes[30] in each analyzed cell, where cells are grouped by automatically annotated cell type based on the reference dataset BaronPancreasData. Marker genes: Ins1 and Ins2 for Beta cells; Gcg for Alpha cells; Sst for Delta cells; Ppy for Gamma cells; Krt19 for Ductal cells; Cpa1, Lyz2, Ptprc for Acinar cells; RGS5 for Activated stellate cells; Sparc, Pecam1 for Endothelial cells.

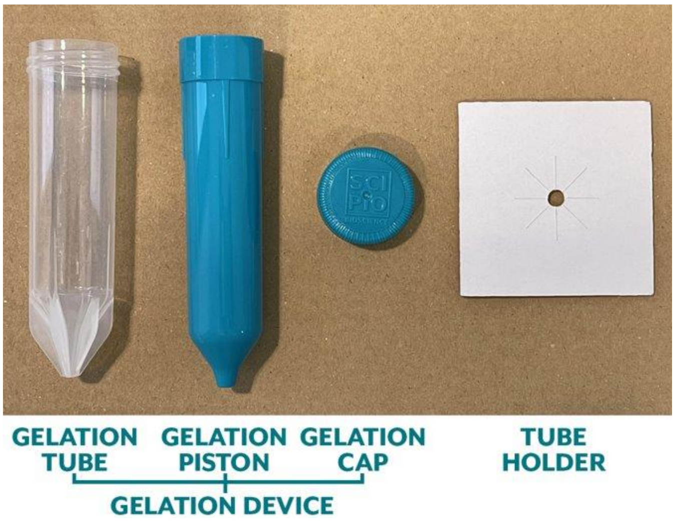

Figure S3. Components of the gelation device: the gelation tube similar in size to a standard 50 mL Falcon conical centrifuge tube, the gelation piston, the gelation cap, and the accompanying tube holder. Spacer guides between the tube and the piston ensure a uniform gel thickness of 500 microns.

Table S1. Oligonucleotide sequence for RevGel-seq

**Oligonucleotide**	**Sequence (5' -> 3')**
Second strand oligo	AAGCAGTGGTATCAACGCAGAGTGANNNGGNNNB
PCR oligo (TS-PCR)	AAGCAGTGGTATCAACGCAGAGT
Custom library primer	AATGATACGGCGACCACCGAGATCTACACGCCTGTCCGCGG AAGCAGTGGTATCAACGCAGAG T*A*C
Custom read1 oligo	GCCTGTCCGCGGAAGCAGTGGTATCAACGCAGAGTAC

*Phosphorothioate bonds to protect from nuclease

Table S2. PCR condition for barcoded cDNA amplification

**Step number and description**		**Number of cycles**	**Temperature**	**Duration**
Step 1	Initial denaturation	-	95°C	3 min
Step 2	Denaturation	4	98°C	20 sec
	Annealing		65°C	45 sec
	Extension		72°C	3 min
Step 3	Denaturation	9	98°C	20 sec
	Annealing		67°C	20 sec
	Extension		72°C	3 min
Step 4	Final extension	-	72°C	5 min
Step 5	Storage	-	4°C	∞
